# Alopecia Universalis Associated with Hyperthyroidism Treated with Azathioprine and Hydroxychloroquine: A Case Report

**DOI:** 10.31729/jnma.5830

**Published:** 2021-09-30

**Authors:** Vikash Paudel, Deepa Chudal, Manish Bhakta Pradhan, Rupa Thakur, Buddhi Raj Pandey

**Affiliations:** 1 National Medical College, Birgunj, Parsa, Nepal; 2 Nepal Police Hospital, Kathmandu, Nepal

**Keywords:** *alopecia areata*, *alopecia universalis*, *azathioprine*, *hydroxychloroquine*

## Abstract

Alopecia universalis is an uncommon form of alopecia areata involving hair loss over the entire scalp and body. This condition is difficult to treat and sustain the growth of hair for longer duration. We report a case of alopecia universalis associated with severe hyperthyroidism. A lady in her fourth decade presented to us with gradual onset of alopecia universalis, who later found to have hyperthyroidism which was refractory to multiple treatment modalities. She was treated successfully with azathioprine and hydroxychloroquine. Alopecia universalis with less response to oral steroid therapy was successfully managed with azathioprine with hydroxychloroquine.

## INTRODUCTION

Alopecia areata (AA) is a common form of multifactorial immune-mediated non-scarring alopecia.^1^ Alopecia universalis (AU) is its rarest variant where all body hairs including eye brows and eyelashes are lost. It might be associated with disorders of autoimmunity like hyperthyroidism, rheumatoid arthritis, lupus erythematosus.^2-3^ Treatment of AA is often difficult because of the lack of effective therapeutic modalities.^4^ We present a case of AU which showed improved outcome with azathioprine and hydroxychloroquine.

## CASE REPORT

**Figure 1A, 1B:**
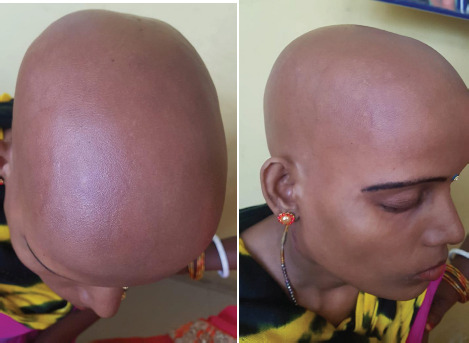
Top view and Lateral view AU showing loss of hair of scalp, eye brows and eye lashes.

A married female of 35 years of age presented to our clinic with a history of gradual loss of all body hairs of a month duration. She first noticed multiple localized patches of sudden loss of the scalp hair which later progressed within a month to cover the entire area of scalp and body with involved eyelashes, eye brows, axillary hairs, and pubic hairs (Figure 1A, 1B).

On examination, she also had exophthalmic eye changes, warm body surface, and heat intolerance but no goitre. As she had clinical features suggestive of hyperthyroidism, it was confirmed after laboratory workup of thyroid hormones and thyroid simulative hormones. Antinuclear antibody was also positive, with a high titre. Anti-thyroid peroxidase antibody was also elevated with negative anti-double stranded DNA.

**Figure 2A, 2B:**
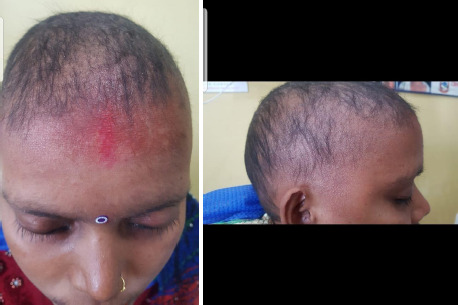
Frontal view and Lateral view of Growth of hair in scalp, eye brow and eye lashes in AU after azathioprine and hydroxychloroquine at 1 month.

The patient was diagnosed as having AU with autoimmune hyperthyroidism and was started a daily oral dose of 40mg of prednisolone and topical steroid flucinolone acetonide, minoxidil 2% and carbimazole. For management of hyperthyroidism, she was referred to an endocrinologist. During treatment of hair loss, eye lashes and eye brows had mild response, but the scalp hair had very minimal response even after six weeks of therapy. As there was very minimal response with the oral and topical steroids, hydroxyl-chloroquine and azathioprine were added along with oral steroids. The oral steroid was stopped after a week to continue azathioprine and hydroxychloroquine. After about a month of stopping steroid and addition of azathioprine and hydroxychloroquine, she started improving with the growth of hairs in the scalp region (Figure 2A, 2B).

She lost follow-up after two months of therapy.

## DISCUSSION

AA is a common form of immune-mediated nonscarring hair loss occurring worldwide. Different types of AA include patchy AA, alopecia sub-totalis, alopecia totalis, and AU.^4^ If the pattern of hair loss involves the entire scalp, it's called alopecia totalis and if the entire scalp and body is involved, it's term as AU.^5^ It occurs equally in men and women, without any preference for race/ethnicity or age. AU has been associated with various autoimmune diseases, most commonly autoimmune like autoimmune thyroiditis diseases, vitiligo, lupus erythematosus, rheumatoid arthritis etc.^3^

Almost half of patients with AA spontaneously recover within 1 year, with or without treatment. However, full recovery from AA without relapse is uncommon, especially with the more severe forms of alopecia totalis and universalis.^4^

Treatment of AA is often difficult and frustrating because of the lack of effective treatment. First-line therapy includes topical and intralesional glucocorticoids, and topical immunotherapy; various second-line treatment options include systemic glucocorticoids, minoxidil, phototherapy, cyclosporine, hydroxychloroquine, sulfasalazine, methotrexate, azathioprine and newer therapies like tofacitinib, biologics.^6-9^

The exact pathogenesis of AA remains unknown, however autoimmune, genetics, environmental factors, and stress are believed to have a role. Histopathology of lesional skin with active disease shows perifollicular (peribulbar) inflammation with lymphocytic infiltrate surrounding anagen follicles (swarm of bees' appearance).^3^ It is believed that CD4+ and CD8+ T cells infiltrate the hair and become reactive to hair bulb autoantigens, leading to inflammation, alterations in hair cycling, and ultimately hair loss. Many other factors have been implicated in the process, including antigen presentation, cytokine release, cytotoxic T-cell activity, and cell death.^4^

Here, AU with hyperthyroidism resistant to oral steroid therapy was successfully treated with azathioprine and hydroxyl-chloroquine and able to maintain the growth of hair till patient was in follow up. Hydroxychloroquine with no immunosuppressive property could be used as an adjuvant in AU.

## References

[1] Simakou T, Butcher JP, Reid S, Henriquez FL (2019). Alopecia areata: A multifactorial autoimmune condition.. J Autoimmun..

[2] Islam N, Leung PS, Huntley AC, Gershwin ME (2015). The autoimmune basis of alopecia areata: a comprehensive review.. Autoimmun Rev..

[3] Alkhalifah A, Alsantali A, Wang E, McElwee KJ, Shapiro J (2010). Alopecia areata update: part I. Clinical picture, histopathology, and pathogenesis.. J Am Acad Dermatol..

[4] Buckley J, Rapini RP (2020). StatPearls [Internet]..

[5] Hordinsky MK (2013). Overview of alopecia areata.. J Investig Dermatol Symp Proc..

[6] Kassira S, Korta DZ, Chapman LW, Dann F (2017). Review of treatment for alopecia totalis and alopecia universalis.. Int J Dermatol..

[7] Delamere FM, Sladden MM, Dobbins HM, Leonardi-Bee J (2008). Interventions for alopecia areata.. Cochrane Database Syst Rev..

[8] Farshi S, Mansouri P, Safar F, Khiabanloo SR (2010). Could azathioprine be considered as a therapeutic alternative in the treatment of alopecia areata? A pilot study.. Int J Dermatol..

[9] Akdogan N, Ersoy-Evans S (2021). Hydroxychloroquine treatment for Alopecia Universalis: Report of six cases.. Australas J Dermatol..

